# The Global Prevalence of Nonalcoholic Fatty Liver Disease and its Association With Cancers: Systematic Review and Meta-Analysis

**DOI:** 10.2196/40653

**Published:** 2023-07-19

**Authors:** Nor Asiah Muhamad, Nur Hasnah Maamor, Fatin Norhasny Leman, Zuraifah Asrah Mohamad, Sophia Karen Bakon, Mohd Hatta Abdul Mutalip, Izzah Athirah Rosli, Tahir Aris, Nai Ming Lai, Muhammad Radzi Abu Hassan

**Affiliations:** 1 Sector for Evidence-based Healthcare National Institutes of Health Ministry of Health Setia Alam Malaysia; 2 Institute for Medical Research National Institutes of Health Ministry of Health Shah Alam Malaysia; 3 Institute for Public Health National Institutes of Health Ministry of Health Shah Alam Malaysia; 4 School of Medicine Taylor’s University Subang Jaya, Selangor Malaysia; 5 Office of the Director General of Health Ministry of Health Putrajaya Malaysia

**Keywords:** fatty liver, nonalcoholic fatty liver, NAFL, nonalcoholic fatty liver disease, NAFLD, prevalence, cancers, cancer, extrahepatic, liver, carcinoma

## Abstract

**Background:**

Nonalcoholic fatty liver disease (NAFLD) is one of the common causes of chronic liver disease globally. Obesity, metabolic diseases, and exposure to some environmental agents contribute to NAFLD. NAFLD is commonly considered a precursor for some types of cancers. Since the leading causes of death in people with NAFLD are cardiovascular disease and extrahepatic cancers, it is important to understand the mechanisms of the progression of NAFLD to control its progression and identify its association with extrahepatic cancers. Thus, this review aims to estimate the global prevalence of NAFLD in association with the risk of extrahepatic cancers.

**Objective:**

We aimed to determine the prevalence of various cancers in NAFLD patients and the association between NAFLD and cancer.

**Methods:**

We searched PubMed, ProQuest, Scopus, and Web of Science from database inception to March 2022 to identify eligible studies reporting the prevalence of NAFLD and the risk of incident cancers among adult individuals (aged ≥18 years). Data from selected studies were extracted, and meta-analysis was performed using random effects models to obtain the pooled prevalence with the 95% CI. The quality of the evidence was assessed with the Newcastle-Ottawa Scale.

**Results:**

We identified 11 studies that met our inclusion criteria, involving 222,523 adults and 3 types of cancer: hepatocellular carcinoma (HCC), breast cancer, and other types of extrahepatic cancer. The overall pooled prevalence of NAFLD and cancer was 26% (95% CI 16%-35%), while 25% of people had NAFLD and HCC (95% CI 7%-42%). NAFLD and breast cancer had the highest prevalence out of the 3 forms of cancer at 30% (95% CI 14%-45%), while the pooled prevalence for NAFLD and other cancers was 21% (95% CI 12%-31%).

**Conclusions:**

The review suggests that people with NAFLD may be at an increased risk of cancer that might not affect not only the liver but also other organs, such as the breast and bile duct. The findings serve as important evidence for policymakers to evaluate and recommend measures to reduce the prevalence of NAFLD through lifestyle and environmental preventive approaches.

**Trial Registration:**

PROSPERO CRD42022321946; https://www.crd.york.ac.uk/prospero/display_record.php?RecordID=321946

## Introduction

Nonalcoholic fatty liver disease (NAFLD) has a global prevalence of 25% and is a leading cause of cirrhosis and hepatocellular carcinoma [1]. NAFLD encompasses a disease continuum from steatosis with or without mild inflammation (nonalcoholic fatty liver; NAFL) to nonalcoholic steatohepatitis (NASH), which is characterized by necroinflammation and faster fibrosis progression than nonalcoholic fatty liver. NAFLD has a bidirectional association with components of the metabolic syndrome, and type 2 diabetes increases the risk of cirrhosis and related complications. It has become one of the most frequent chronic liver diseases. NAFLD is determined by the presence of ≥5% in the liver or more of hepatic steatosis [2] in liver magnetic resonance imaging proton density fat fraction findings or biopsies in the absence of secondary causes of hepatic fat accumulation, such as hepatitis C infection and glycogen storage disease [3]. At the same time, other approaches to diagnose NAFLD, such as clinical and laboratory scores, have been developed, even though the accuracy cannot be determined. Several biomarkers demonstrate better performance in the diagnosis of NAFLD. Therefore, many approaches are being combined with artificial intelligence to increase diagnostic performance [4]. Electronic health education, which includes the use of mobile communication devices (eg, smartphones and tablet computers), creates awareness and health alerts regarding the disease [5]. This technique is useful for educational purposes, such as promoting healthy behavior for community prevention and early screening.

NAFLD encompasses a spectrum of diseases including NAFL, which has a more benign course, and NASH, which can progress to cirrhosis and hepatocellular carcinoma (HCC) [6]. Due to the heterogeneous nature of the disease, the undetermined symptoms, and the high disease burden, there is increasing appreciation that NAFLD may also be becoming an important cause of HCC [7]. The reported global prevalence of NAFLD and HCC varies between 2% and 58.5% [8].

NAFLD is a complex, multifactorial disease caused by a sedentary lifestyle, obesity, poor dietary habits, intestinal flora, genetics, and other factors [9-11]. NAFLD typically occurs in patients with metabolic syndrome, ranging from simple hepatic steatosis to nonalcoholic steatohepatitis [12]. Visceral adiposity and insulin resistance are among important conditions associated with NAFLD that have been extensively studied [13-17]. Although NAFLD normally has a good prognosis, it can progress to nonalcoholic steatohepatitis, liver fibrosis, cirrhosis, HCC, and even breast cancer [18-20], with male patients having a high risk of such complications [21]. The presence of progressive liver disease is frequently detected only after it has advanced to a late stage. Patients with advanced liver disease usually do not respond well to intervention and have high risks of mortality and morbidity. The prevalence of NAFLD and its association with hepatic and extrahepatic complications have been reported in individual studies, many of which have been published recently. An up-to-date review of the current evidence to determine the global magnitude of the problem is warranted.

In this review, we systematically synthesized published online evidence on the global prevalence of NAFLD and quantified the magnitude of the association between NAFLD and the risk of extrahepatic cancers.

## Methods

We conducted a systematic review of observational studies and report our findings in accordance with the PRISMA (Preferred Reporting Items for Systematic Reviews and Meta-Analyses) guidelines (Multimedia Appendix 1) and the GATHER (Guidelines for Accurate and Transparent Health Estimates Reporting) statement [22] (Multimedia Appendix 2).

### Search Strategy

We searched various electronic databases, including PubMed, ProQuest, Scopus, and Web of Science, from inception to March 31, 2022, to identify relevant studies reporting the prevalence of NAFLD and cancers incident among adults (aged ≥18 years). A search strategy was generated systematically for PubMed and adapted for use in the other electronic databases. Keywords and comparable medical subject heading (MESH) terms were used in combination where appropriate, without restriction on language or publication year. Specific search terms were as follows: (“Fatty liver” OR “steatosis”) AND (“cancer” OR “malignancy” OR “tumour” OR “carcinoma”) AND “prevalence” for the PubMed search. Searches were restricted to human studies. Studies in languages other than English were excluded. Additionally, we screened cross-references from relevant original papers and review articles to identify primary studies not covered by the original database searches. We used a reference manager (EndNote; Clarivate Plc) to store all the studies and citations and developed a database for the included and excluded studies. We integrated our reporting with the MOOSE (Meta-Analysis of Observational Studies in Epidemiology) guidelines [23].

### Criteria for Considering Studies for This Review

We included all observational studies that evaluated the prevalence of NAFLD and examined the association between NAFLD and the risk of developing cancers (Textbox 1). We included studies with an adult population aged 18 years and older of either sex without any restriction in terms of race or ethnicity in the meta-analysis. We excluded conference abstracts, case reports, reviews, commentaries, editorials, practice guidelines, and cross-sectional studies. In the case of multiple studies using the same cohort, the study with the most detailed information on the participants or the largest number of participants was selected. We included studies according to the population, intervention, comparator, outcome, study (PICOS) approach (Table S1 in Multimedia Appendix 3).

**Inclusion and exclusion criteria.**

**Inclusion criteria**

- Observational study
- Participants were adults aged 18 years and older

**Exclusion criteria**

- Conference abstracts, case reports, reviews, commentaries, editorials, practice guidelines, and cross-sectional studies
- Participants were younger than 18 years

### Study Selection

Two authors (NHM and FNL) independently screened all the titles and abstracts to search for potential studies identified as a result of the search and coded them as “retrieve” (eligible, potentially eligible, or unclear) or “do not retrieve.” Another 2 authors (ZAM and SKB) independently retrieved the full-text study reports and publications to identify studies for inclusion, as well as identify and record reasons for the studies’ exclusion. In case of discrepancies, an agreement was reached by consensus and discussion with a third reviewer (NML).

### Data Extraction Process

Two authors (IAR and MHAM) independently evaluated the methodological quality of each included study and extracted data using an electronic form in the reference manager, which was adapted from the Cochrane Handbook for Systematic Review of Interventions [24]; discrepancies were resolved by discussion with a third author (NAM). A standardized data collection form was used to extract the data. The extraction form contained information about the author, year of publication, setting (country of origin), population, study design, sample size, study period, presence of fatty liver, method of detection of fatty liver, type of cancer, and prevalence of NAFLD.

### Risk of Bias in Included Studies

Methodological quality was determined using a domain-based tool adapted from the Newcastle-Ottawa Scale (NOS) to assess the risk of bias of each study (Multimedia Appendix 4). We classified the risk of bias as either low, moderate, high, or unclear across the following domains: selection of participants (selection bias), sample size justification (selection bias), outcome measurement (detection bias), and confounding adjustment. We assigned a score of 7 and above as good quality, and below 6 as having concerns related to determining the overall quality [25].

### Data Synthesis and Analysis

We used Stata (version 16; StataCorp) for all statistical analysis. The pooled prevalence rates, as well as their 95% CIs, were calculated using a random effects model [26]. The *I*^2^ statistic and Cochran *Q* test were used to assess heterogeneity among the studies [27]. The *I*^2^ describes the percentage of the variability in effect estimates that is attributable to heterogeneity rather than sampling error. A value greater than 50% may be considered to indicate substantial heterogeneity, whereas a score of more than 75% indicates high heterogeneity [26,28].

## Results

### Identification of Studies

**Figure 1:**
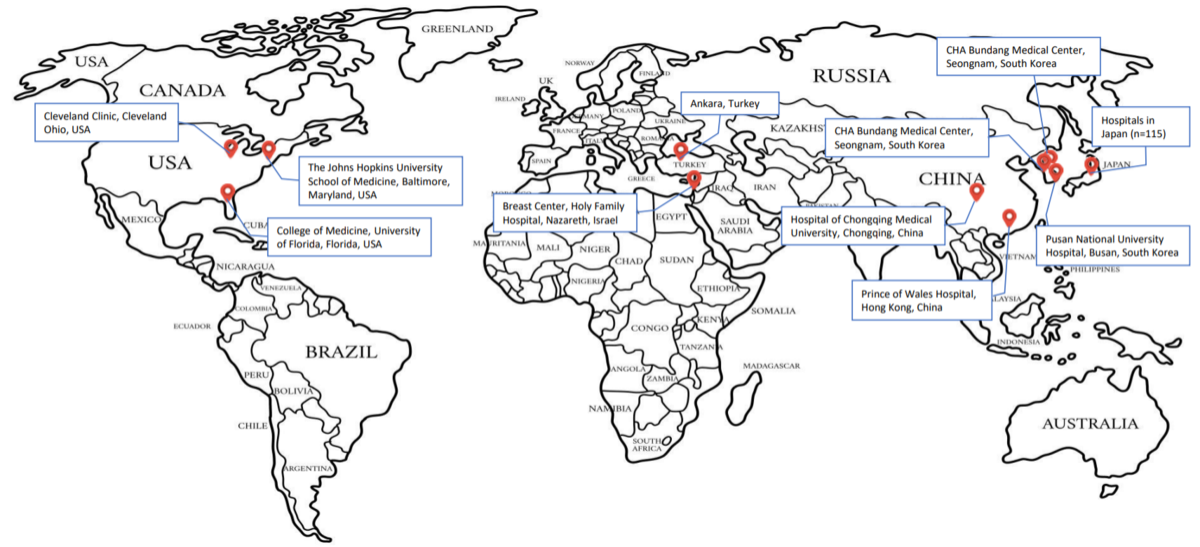
Map of study sites in the included articles.

**Figure 2:**
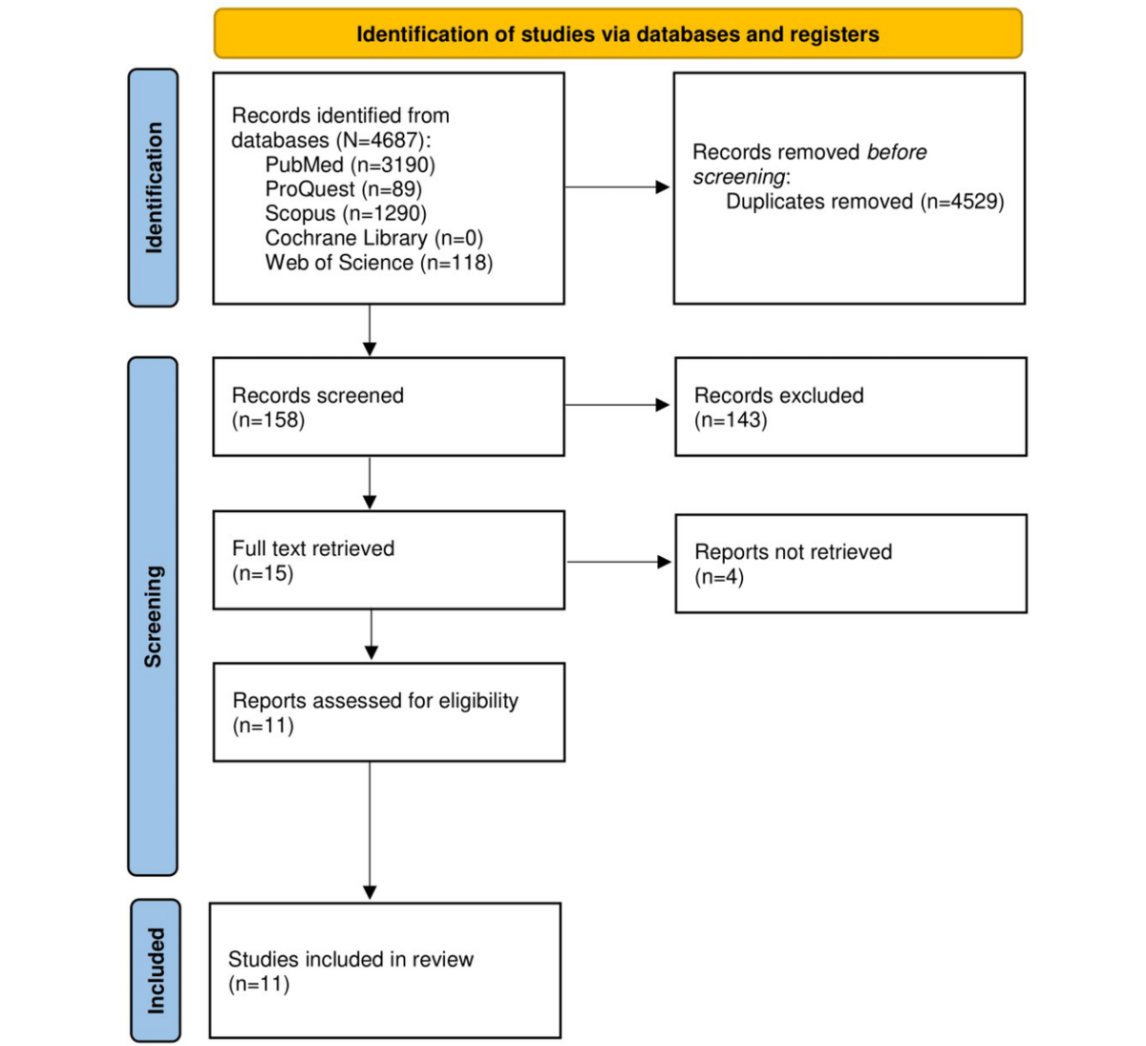
PRISMA (Preferred Reporting Items for Systematic Reviews and Meta-Analyses) flow diagram of the selection process.

From the preliminary search, we identified 4687 studies across all databases, including PubMed (n=3190), ProQuest (n=89), Scopus (n=1290), and Web of Science (n=118). After removing duplicates, we screened a total of 158 titles and abstracts, of which 143 were excluded. We retrieved a total of 15 full texts for inclusion. We excluded 4 studies from the full texts and included 11 studies that met our inclusion criteria for this review. The most common reasons for exclusion were (1) no data on population prevalence of NFALD and cancer was reported and (2) the cancer condition was not included in the study. After removing duplicates, titles, and abstracts, 4 studies were excluded. We included 11 studies, as shown in Table 1 and Figure 1. Figure 2 shows the selection process to complete the PRISMA flow diagram.

**Table 1:** Characteristics of included studies.

Author/year	Country	Study design	Sample size, n	Setting	NAFLD^a^ diagnosis	Type of cancer	Length of follow up	Prevalence of NAFLD (%)	Hazard ratio (95% CI)
Başaranoğlu et al (2014) [29]	Turkey	Prospective	105	Hospital	NAFLD defined by observing increased echogenicity and liver-kidney contrast using ultrasonography	Cancer in first-degree relatives	Not stated	27	Not stated
Lee et al (2017) [30]	Korea	Retrospective	104	Hospital	NAFLD defined by observing hepatic fat accumulation using magnetic resonance imaging	Breast	Not stated	18.3	Not stated
Chan et al (2017) [31]	China	Retrospective	270	Hospital	NAFLD defined as presence of steatosis ≥5%; steatohepatitis was defined as the presence of steatosis ≥5%, ballooning degeneration, and lobular inflammation in a liver biopsy	HCC^b^	3-12 months	39.6	6.84 (1.48-31.66)
Tian et al (2021) [32]	China	Cohort	263	Hospital	NAFLD investigated by ultrasound. Ten successful reads were required and the median was recorded. The ratio of the IQR divided by median (IQR/median) of all measurements less than 30% with a success rate (successful tests/total tests) ≥60% was regarded as a valid measurement and controlled attenuation parameter ≥240 was defined as hepatic steatosis	Breast	Not stated	41.8	Not stated
Tokushige et al (2011) [33]	Japan	Cohort	292	Hospital	Diagnosis of NAFLD was based on the following criteria: (1) detection of hepatic steatosis (or steatohepatitis) by liver biopsy or imaging; (2) intake of less than 20-30 grams of ethanol per day (as confirmed by the attending physician and family members who were in close contact with the patient); and (3) appropriate exclusion of other liver diseases (such as alcoholic liver disease, viral hepatitis, autoimmune hepatitis, drug-induced liver disease, primary biliary cirrhosis, primary sclerosing cholangitis, biliary obstruction, and metabolic liver diseases such as Wilson disease and hemochromatosis).	HCC	Not stated	2.0	Not stated
Lee et al (2019) [34]	Korea	Cohort	321	Hospital	NAFLD defined as the presence of ≥5% hepatic steatosis using ultrasound	HCC	3-6 months	8.2	3.005 (1.122- 8.051)
Zarrinpar et al (2019) [35]	US	Retrospective	317	Hospital	NAFLD defined by having steatohepatitis based on the hospital file record	HCC	Not stated	24.0	Not stated
Reddy et al (2013) [36]	US	Cohort	181	Hospital	The underlying liver pathology reported in this study was based on a dedicated rereview of the pathological slides of each resection specimen by an experienced hepatobiliary pathologist at each respective center. Steatosis grade, lobular inflammation, hepatocyte ballooning, extent of fibrosis, and portal inflammation were described according to previous articles. Instead of the precise number of foci per high power field, lobular inflammation was reported as “none,” “rare/spotty,” “mild,” or “moderate/heavy.” Each of these terms was then coded in increasing severity from 0 to 3 to calculate the NAFLD activity score.	Intrahepatic; cholangiocarcinoma	Not stated	17.1	Not stated
Nseir et al (2017) [37]	Israel	Retrospective	133	Hospital	Presence of hepatic steatosis on abdominal computerized tomography examination and an attenuation of –5 to 10 Hounsfield units (calculated as liver attenuation minus spleen attenuation), no alcohol consumption (<20 g/day), negative serology for hepatitis B or C virus, negative to antibodies for autoimmune hepatitis, and no history of other known liver disease	Breast	Not stated	45.2	Not stated
Asfari et al (2020) [38]	US	Cross-sectional	218,950	Hospital	The study group was identified using the International Classification of Diseases 9th version code for nonalcoholic steatohepatitis	HCC	Not stated	50.0	Not stated
Lee et al (2019) [39]	Korea	Retrospective	1587	Hospital	NAFLD was diagnosed when the mean attenuation of the liver was lower than 40 Hounsfield units or 10 units lower than that of the spleen by using CT scanning, magnetic resonance imaging, or biopsy	Breast	Not stated	15.8	1.581 (1.038-2.410)

^a^NAFLD: nonalcoholic fatty liver disease.

^b^HCC: hepatocellular carcinoma.

### Characteristics of Included Studies

A total of 11 studies with 222,523 adult participants (aged ≥18 years) across 6 countries were included. A total of 3 studies were conducted in the United States and 8 studies were from 5 countries in Asia: South Korea (n=3), China (n=2), Turkey (n=1), Japan (n=1), and Israel (n=1). Cancer was classified into 3 types: HCC (5 studies), breast cancer (4 studies), and others (2 studies). The characteristics of the included studies are depicted in Table 1.

### Risk of Bias of Included Studies

We assessed the quality of the included studies [29-39] as NOS quality, with modifications as shown in Table S2 in Multimedia Appendix 3. All 11 studies were observed to have good quality (NOS score 7 and above).

### The Estimates of Pooled Prevalence of NAFLD and Cancer

**Figure 3:**
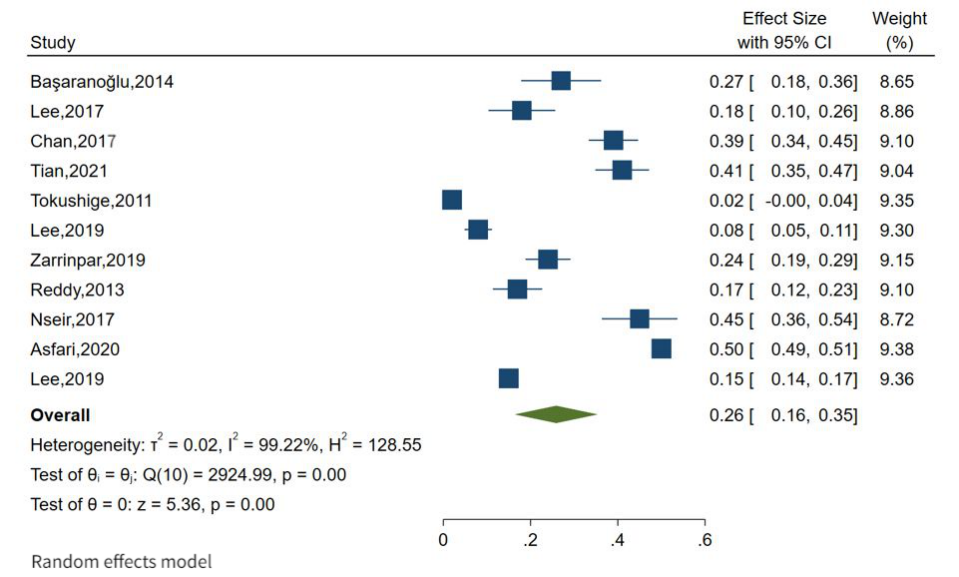
Forest plot of overall prevalence of fatty liver and cancer [29-39].

**Figure 4:**
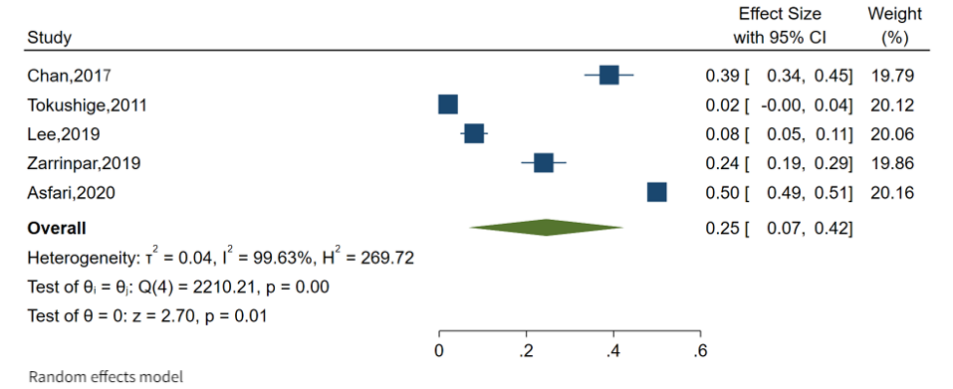
Forest plot of overall prevalence of fatty liver and hepatocellular carcinoma [31,33-35,38].

**Figure 5:**
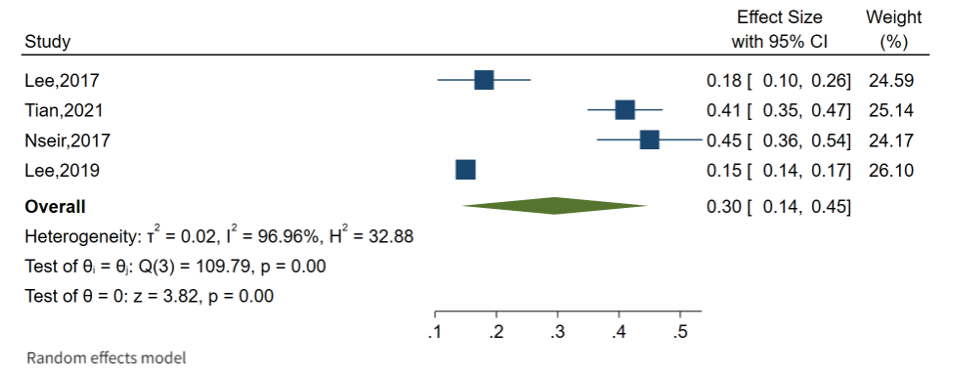
Forest plot of overall prevalence of fatty liver and breast cancer [30,32,37,39].

**Figure 6:**
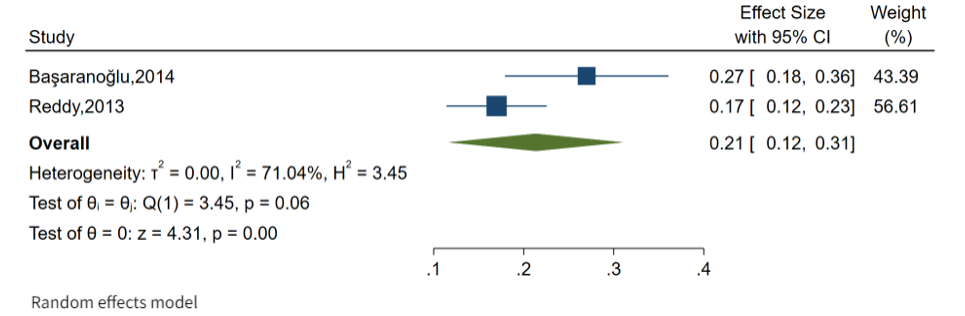
Forest plot of overall prevalence of fatty liver and other cancers [29,36].

The overall pooled prevalence of NAFLD among the adult population was 26% (95% CI 16%-35%), as shown in Figure 3, with a high level of heterogeneity between studies (*I*^2^=99.2%; *P*=.001). The overall pooled prevalence of HCC was calculated based on these findings, and it was observed that the overall pooled prevalence of HCC was 25% (95% CI 7%-42%) with a high level of heterogeneity between studies (*I*^2^=99.6%; *P*=.001) reported in Asia (4 publications) and the United States (1 publication; Figure 4). The overall pooled prevalence of breast cancer was 30% (95% CI 14%-45%) with a high level of heterogeneity between studies (*I*^2^=96.9%; *P*=.001) reported in Asia (4 publications), as shown in Figure 5. In this review, cancer in first degree relatives and intrahepatic cholangiocarcinoma showed an overall pooled prevalence of 21% (95% CI 12%-31%) with a moderate level of heterogeneity between studies (*I*^2^=71%; *P*=.001) reported in Asia and the United States, each with 1 publication (Figure 6).

## Discussion

### Principal Findings

This review elucidates an assessment of NAFLD as a worldwide epidemic that may contribute to chronic liver disease. In this systematic review and meta-analysis, we included 11 studies to comprehensively estimate the global prevalence and the association with cancers among the adult population with NAFLD. There is strong evidence that suggests the association of NAFLD with various diseases, such as cardiovascular disease, diabetes, and cancers such as bile duct, breast, and liver cancer [11,40]. In recent years, more research and discussion has been focused on the possible association between NAFLD and the risk of cancers. Therefore, this review will be useful in expounding the latest research progress on this issue [41].

In this review, the overall pooled prevalence of NAFLD and cancers was 26%. The pooled prevalence of NAFLD and breast cancer was higher than that of HCC and other cancers (30% vs 25% and 21%, respectively). This review also discovered that the highest prevalence of NAFLD is reported in breast cancer, which is consistent with the findings of studies by Kim et al [42] (2017) and Mantovani et al [43] (2022). According to the former report, NAFLD showed a strong association with the development of HCC and colorectal cancer in men and breast cancer in women [42]. The latter study, a meta-analysis, examined the risk of several prespecified cancers and found an increased risk of developing breast cancer [43].

A review by Thomas et al [44] (2022) examined NAFLD and the incidence of hepatic and extrahepatic cancers. The review found that the pooled incidence rate of HCC was 1.25 per 1000 person-years. However, our review examined the prevalence of HCC among the adult population with NFLD. Another review [45] found that the presence of NAFLD was independently associated with an 88% increased risk of HCC, as compared to the absence of NAFLD. This review is different from ours, which examined the prevalence of HCC with NAFLD.

Although the exact or detailed mechanism of this interaction remains unclear, cancer development in patients with NAFLD may be associated with a bidirectional interaction between NAFLD and metabolic syndrome [39]. According to Tiniakos et al [46] (2018), the increased susceptibility of the liver with steatosis to carcinogenic insults could be linked to metabolic derangements, such as metabolic syndrome, hyperinsulinemia, and the presence of insulin-like growth factor receptors in HCC, as well as systemic effects of deranged cytokines and adipokines, immune dysregulation, and changes in gut microbiota. Besides that, the genetic component has been named as another factor that contributes to an increased risk of HCC in individuals with NAFLD [46].

A few mechanisms for extrahepatic carcinogenesis of the fatty liver, such as breast cancer, have been proposed. First, high levels of inflammatory cytokines are closely associated with NAFLD, as they promote insulin resistance and elevated circulating triglycerides, influence growth, and increase apoptosis and tumor cell proliferation in many cancers [47]. Second, hyperinsulinemia and high levels of leptin have carcinogenic effects [48]. By binding to the circulating sex hormone–binding globulin, increasing insulin levels cause the elevated secretion of estrogen, and downstream signaling favors breast carcinogenesis [49]. Insulin may cross-bind to insulin-like growth factor-I (IGF-1) receptors on breast cells, and downstream signaling pathways provide proliferation stimuli to breast cancer cells [19]. Third, decreased levels of adiponectin lead to marked insulin resistance and subsequent increased levels of IGF-1. Insulin binds to IGF-1 receptors and plays an important role in cell proliferation, apoptosis, and increased production of vascular endothelial growth factors [37].

### Strengths and Study Limitations

This study has several strengths. To our knowledge, this is a comprehensive and up-to-date systematic review and meta-analysis that reports the pooled prevalence of NAFLD and cancers. We examined a number of studies using stringent inclusion criteria. Following the broad search strategy, we were able to stratify hepatic and extrahepatic cancers. There are potential limitations in our review. The included studies largely had an observational design, and there are risks for a number of biases in term of design, selection of respondents, and sample size. The included studies were located only in Asia (n=8) or the United States (n=3), limiting the accuracy of the estimations. There is a paucity of data from other countries, and most studies are conducted among the urban population. Therefore, our findings might be biased and have limited generalizability beyond the countries included in this review. In short, there is a need to investigate NAFLD and cancer across different regions in the future.

### Conclusion

This systematic review and meta-analysis shows that the pooled prevalence of NAFLD is closely associated with hepatic and extrahepatic cancers. This review provides evidence that a substantially high proportion of patients with NAFLD are associated with extrahepatic and hepatocellular carcinoma. However, the evidence remains inconclusive, and further studies are needed to confirm the association between NAFLD and cancers, as well as to improve surveillance strategies for patients with NAFLD who are at high risk of cancers. Since the global prevalence of NAFLD is increasing, policymakers must work toward reversing the current trends by increasing the awareness of NAFLD and promoting healthy lifestyles and environments.

## Appendix

Multimedia Appendix 1 PRISMA (Preferred Reporting Items for Systematic Reviews and Meta-Analyses) checklist.

Multimedia Appendix 2 GATHER (Guidelines for Accurate and Transparent Health Estimates Reporting) checklist.

Multimedia Appendix 3 Table S1: The population, intervention, comparator, outcome, study (PICOS) approach. Table S2: Newcastle-Ottawa Quality Assessment Form for Cohort Studies.

Multimedia Appendix 4 Newcastle-Ottawa Quality Assessment Form for Cohort Studies.

## Abbreviations

GATHER: Guidelines for Accurate and Transparent Health Estimates
HCC: hepatocellular carcinoma
IGF-1: insulin-like growth factor-I
MESH: medical subject heading
MRI-PDFF: magnetic resonance imaging proton density fat fraction
NAFL: nonalcoholic fatty liver
NAFLD: nonalcoholic fatty liver disease
NASH: nonalcoholic steatohepatitis
NOS: Newcastle Ottawa Scale
PICOS: population, intervention, comparator, outcome, study
PRISMA: Preferred Reporting Items for Systematic Reviews and Meta-Analyses

## Data Availability

All data generated or analyzed during this study are included in this published article and its supplementary information files.
