# Supplementary material for: The Global Prevalence of Nonalcoholic Fatty Liver Disease and its Association With Cancers: Systematic Review and Meta-Analysis
Source: Interact J Med Res. 2023 Jul 19;12:e40653. doi: 10.2196/40653 (PMC10398554; doi:10.2196/40653)
Supplement: Multimedia Appendix 3 [file ijmr_v12i1e40653_app3.docx]

**Multimedia Appendix 3.**

**Table S1:** PICOS

| **P** | Participant | Individuals with fatty liver incidence and cancer. |
| --- | --- | --- |
| **I** | Intervention | Any component of prevalence in fatty liver incidence and cancer. |
| **C** | Comparator | Any component without prevalence in fatty liver incidence and cancer. |
| **O** | Outcome | Incidence or prevalence of fatty liver and cancer. |
| **S** | Study | Any observational studies except commentaries, editorial, case series and systematic review. |

**Table S2**: Newcastle-Ottawa Quality Assessment Form for Cohort Studies

| **No** | **Author/year** | **Selection** | | | | **Comparability** | **Outcome** | | | **Quality score** |
| --- | --- | --- | --- | --- | --- | --- | --- | --- | --- | --- |
|  |  | **S1** | **S2** | **S3** | **S4** |  | **O1** | **O2** | **O3** |  |
| 1 | Lee et al. (2019) [39] | * | * | * | * | * |  | * | * | Good |
| 2 | Asfari et al. (2020) [38] | * | * | * | * | * |  | * | * | Good |
| 3 | Nseir et al. (2017) [37] | * | * | * | * | * |  | * | * | Good |
| 4 | Reddy et al (2013) [36] | * | * | * | * | * |  | * | * | Good |
| 5 | Zarrinpar et al (2019) [35] | * | * | * | * | * |  | * | * | Good |
| 6 | Lee et al. (2019) [34] | * | * | * | * | * |  | * | * | Good |
| 7 | Tokushige et al. (2011) [33] | * | * | * | * | * |  | * | * | Good |
| 8 | Tian et al. (2021) [32] | * | * | * | * | * |  | * | * | Good |
| 9 | Chan et al. (2017) [31] | * | * | * | * | * |  | * | * | Good |
| 10 | Lee et al. (2017) [30] | * | * | * | * | * |  | * | * | Good |
| 11 | Başaranoğlu et al. (2014) [29] | * | * | * | * | * |  | * | * | Good |
