# Supplementary material for: The Global Prevalence of Nonalcoholic Fatty Liver Disease and its Association With Cancers: Systematic Review and Meta-Analysis
Source: Interact J Med Res. 2023 Jul 19;12:e40653. doi: 10.2196/40653 (PMC10398554; doi:10.2196/40653)
Supplement: Multimedia Appendix 4 [file ijmr_v12i1e40653_app4.docx]

**Multimedia Appendix 4.**

**Newcastle-Ottawa Quality Assessment Form for Cohort Studies**

Note: A study can be given a maximum of one star for each numbered item within the Selection and Outcome categories. A maximum of two stars can be given for Comparability.

# Selection

1. Representativeness of the exposed cohort
   1. Truly representative ***(one star)***
   2. Somewhat representative ***(one star)***
   3. Selected group
   4. No description of the derivation of the cohort
2. Selection of the non-exposed cohort
   1. Drawn from the same community as the exposed cohort ***(one star)***
   2. Drawn from a different source
   3. No description of the derivation of the non exposed cohort
3. Ascertainment of exposure
   1. Secure record (e.g., surgical record) ***(one star)***
   2. Structured interview ***(one star)***
   3. Written self report
   4. No description
   5. Other
4. Demonstration that outcome of interest was not present at start of study
   1. Yes ***(one star)***
   2. No

# Comparability

1. Comparability of cohorts on the basis of the design or analysis controlled for confounders
   1. The study controls for age, sex and marital status ***(one star)***
   2. Study controls for other factors (list) ***(one star)***
   3. Cohorts are not comparable on the basis of the design or analysis controlled for confounders

# Outcome

1. Assessment of outcome
   1. Independent blind assessment ***(one star)***
   2. Record linkage ***(one star)***
   3. Self report
   4. No description
   5. Other
2. Was follow-up long enough for outcomes to occur
   1. Yes ***(one star)***
   2. No

Indicate the median duration of follow-up and a brief rationale for the assessment above:

1. Adequacy of follow-up of cohorts
   1. Complete follow up- all subject accounted for ***(one star)***
   2. Subjects lost to follow up unlikely to introduce bias- number lost less than or equal to 20% or description of those lost suggested no different from those followed. ***(one star)***
   3. Follow up rate less than 80% and no description of those lost
   4. No statement

## Thresholds for converting the Newcastle-Ottawa scales to AHRQ standards (good, fair, and poor):

**Good quality:** 3 or 4 stars in selection domain AND 1 or 2 stars in comparability domain AND 2 or 3 stars in outcome/exposure domain

## **Fair quality:** 2 stars in selection domain AND 1 or 2 stars in comparability domain AND 2 or 3 stars in outcome/exposure domain

**Poor quality:** 0 or 1 star in selection domain OR 0 stars in comparability domain OR 0 or 1 stars in outcome/exposure domain
